# Enhancing Structural and Thermal Properties of Poly(lactic acid) Using Graphene Oxide Filler and Anionic Surfactant Treatment

**DOI:** 10.3390/molecules28186442

**Published:** 2023-09-05

**Authors:** Selsabil Rokia Laraba, Najeeb Ullah, Amirouche Bouamer, Asmat Ullah, Tariq Aziz, Wei Luo, Wahiba Djerir, Qurat ul Ain Zahra, Amine Rezzoug, Jie Wei, Yulin Li

**Affiliations:** 1 Key Laboratory for Ultrafine Materials of Ministry of Education, East China University of Science and Technology, Shanghai 200237, China; y10190007@mail.ecust.edu.cn (S.R.L.); kevin0713@126.com (W.L.); 2 Department of Chemical Engineering, University of Tennessee, Chattanooga 615 McCallie Ave., Chattanooga, TN 37403, USA; 3 Research Center in Industrial Technologies (CRTI), P.O. Box 64, Cheraga 16014, Algeriaa.rezzoug@crti.dz (A.R.); 4 Clinical Research Institute, Zhejiang Provincial People’s Hospital, Hangzhou 310014, China; 5 Faculty of Civil Engineering and Mechanics, Jiangsu University, Zhenjiang 212013, China; 6 Biomedical Imaging Center, University of Science and Technology of China, Hefei 230026, China

**Keywords:** poly(lactic acid), graphene oxide, AOT treatment, crystallinity, thermal properties, wettability

## Abstract

Graphene has attracted extensive attention in various fields due to its intriguing properties. In this work, nanocomposite films based on poly(lactic acid) (PLA and PLLA) polymers filled with graphene oxide (GO) were developed. The impact of treating GO with the anionic surfactant dioctyl sulfosuccinate sodium salt (AOT) on the properties of the resulting nanocomposites was investigated. To determine the morphological, optical, and structural properties of the obtained materials, physicochemical analyses were performed, including scanning electron microscopy (SEM), atomic force microscopy (AFM), Fourier-transform infrared spectroscopy (FT-IR), and X-ray diffraction (XRD) analysis. Additionally, the thermal properties and wettability of neat polymers and nanocomposites were thoroughly investigated using differential scanning calorimetry (DSC), thermogravimetric analysis (TGA), and contact angle analysis. It was observed that GO was well dispersed throughout the PLA and PLLA matrix, leading to stronger interface bonding. The results demonstrate that the untreated and treated GO improved the crystallinity and thermal stability properties of the PLA and PLLA. However, the AOT-treated GO has significantly higher performance compared to the untreated GO in terms of crystallinity, melting temperature (increased by ~15 °C), and wettability (the contact angle decreased by ~30°). These findings reveal the high performance of the developed novel composite, which could be applied in tissue engineering as a scaffold.

## 1. Introduction 

Recently, polymer-based biomaterial implants have shown increased use in the biomedical field. They must possess good mechanical properties, nontoxicity, biocompatibility, biodegradability, nonimmunogenicity, and satisfactory tribological performance. Since biocomposites are made by combining natural and synthetic materials, they have an advantage in biocompatibility, tuneability, and biodegradability compared to other polymer-based composites [1,2]. This reduces the impact of therapies and medical devices on the environment and improves patient outcomes [3]. Due to these advantages, researchers are actively developing new biocomposites, which could have promising applications in drug delivery, tissue engineering, and implantable medical devices [4]. 

One of the most attractive materials for biocomposite development is poly(lactic acid) (PLA) [5]. It can be derived from renewable resources like sugarcane, cornstarch, wheat, and other natural materials [6]. Some of its excellent properties include compatibility, low toxicity, and the ability to break down into natural compounds over time. In addition, PLA also shows remarkable processability and high optical transparency. Environmentally, it has reduced the impact of plastic waste in oceans and landfills [7,8]. This aspect has made it an attractive alternative in 3D printing, drug delivery, tissue engineering, and various other biomedical applications. Moreover, PLA uses 25–55% less energy to produce than petroleum-based polymers and is inexpensive compared to petroleum products [9,10,11]. Despite its benefits, there are some disadvantages associated with its properties. Indeed, PLA presents limitations such as its limited crystallization rate, low thermal stability, lack of intrinsic bioactivity, relatively low oxygen barrier, and poor mechanical properties. Consequently, these disadvantages significantly restrict the utilization of PLA in biomedical applications [12]. To address these constraints, researchers have explored the use of graphene and its derivatives as nano-filler material for this polymer [13,14]. 

In recent years, graphene oxide (GO) has emerged as a potential nano-filler material for polymers. In most studies, biodegradable polymers reinforced with GO are PLA, PVA, PCL, and PMMA [15]. In this context, our recent review paper extensively discussed the advantages of incorporating graphene and graphene-related materials into polymers, which include enhancements in mechanical, thermal, electrical, impermeability, and biological properties [16]. GO enhances the mechanical properties of composite materials due to its ability to transfer loads and interact at interfaces due to functional groups. Regarding thermal enhancement, graphene oxide (GO) has remarkable thermal stability and acts as a barrier to prevent the transfer of combustion gases. In terms of electrical conductivity, GO has numerous contact possibilities owing to its 2D character and higher surface area, which play important roles in improving the electrical properties of polymers [16,17,18]. On the other hand, GO and its derivatives show good properties as biomaterials, like biocompatibility, low toxicity, and antibacterial activity. In fact, GO is cytocompatible both in vitro and in vivo due to its ability to interact with biological molecules. Additionally, GO exhibits antioxidant activity owing to the presence of hybridized sp^2^ carbons that can form radical adducts and participate in electron transfer reactions. This aspect makes polymer-based composites reinforced with GO promising candidates for developing biomaterials [19,20].

In this regard, PLA/GO nanocomposites have shown significant interest as biomaterials. For instance, Gu et al. reported that a progressive addition of GO to PLA/PBC nanofibrous membranes resulted in a gradual increase in antibacterial activity, which was attributed to the improved bacteriostatic effect of GO [21]. In support, An et al. observed that incorporating GO into PLA/polyurethane significantly increased its antibacterial activity against S. aureus and E. coli, which is a behavior attributed to GO’s ability to prevent bacteria from growing and adhering to surfaces [22]. This suggests that GO acts as a barrier to prevent bacterial colonization [15]. On the other hand, Amiryaghoubi et al. investigated the use of polymer–graphene hybrid to develop scaffolds for microvascular tissue engineering and regeneration. They studied various synthetic and natural biodegradable polymers and hence identified PLLA as a promising matrix for this specific application [23]. Similarly, Yan et al. [24] effectively obtained GO/PLLA nanofiber scaffolds exhibiting a hydrophilic surface and porous network structure that facilitate cell infiltration. This material holds promise for utilization in ovarian tissue cryopreservation and transplantation [24]. Simultaneously, the incorporation of GO into PLA also enhances the mechanical and surface properties of PLA and hence increases its functionality, including an increased Young’s modulus and tensile strength [15], and it was found to increase the crystallinity of the composite material [25]. 

To prepare a graphene-based composite, there are a set of requirements that need to be fulfilled. This includes (i) a well-dispersed graphene filler within the matrix, (ii) a strong interface binding between the graphene and matrix, and (iii) in specific applications, continuous graphene sheets with a large area or interconnected graphene sheets [16,26]. For this reason, many studies focus on improving the compatibility between the GO filler and PLA matrix within the composites. For instance, Zhang et al. significantly improved the interfacial adhesion between the filler (GO) and matrix PLA by surface grafting GO with poly(ethylene glycol) (PEG). Therefore, the purpose was to prepare nanofibrous composite scaffolds [27]. Valapa et al. revealed that the sonication time of GO has a great influence on its dispersion capability in the PLA matrix [28]. On the other hand, to address the issue of the aggregation of GO within the PLA matrix, Wang et al. grafted GO with L-lactic acid monomer, which was used as filler material for PLLA scaffolds. The results show a uniform dispersion of GO and hence an enhanced performance of the resulting composite [29]. Furthermore, Li et al. improved the interface of PLA blends by using a PLLA-functionalized GO. The grafted PLLA chains enhance the interface interaction of immiscible PLLA/PPC blends by facilitating the formation of a network-like structure [30]. These research results clearly indicate that the compatibility (adhesion and dispersion) of GO with PLA is significantly challenging and could be further improved.

To address this issue, this work is devoted to a surface treatment method applied to GO in order to improve the performance of PLA and PLLA-based nanocomposites. Anionic surfactant dioctyl sulfosuccinate sodium salt (AOT) was chosen in this investigation due to its effect on the dispersibility and thermal stability of GO. Treated and untreated GO-based nanocomposites were compared in terms of crystallinity, thermal stability, and surface wettability. The results of this research can be used to support the future development of PLA/GO-based scaffolds, which are highly required in tissue engineering.

## 2. Results and Discussion 

**Figure 1:**
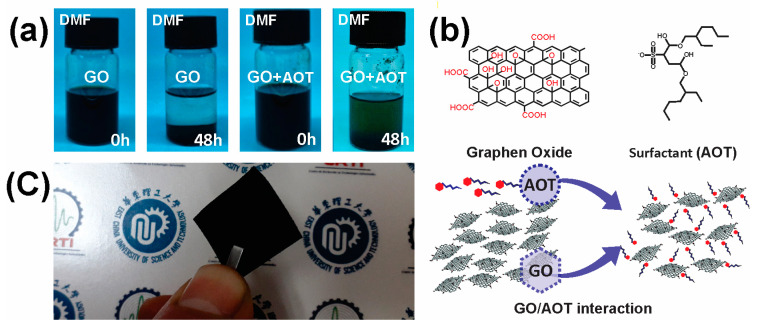
Preparation of the nanocomposites. (**a**) Digital photographs of untreated and treated GO dispersion in DMF solvent at the initial time and after standing for 48 h. (**b**) Schematic diagram of the interaction of AOT with the GO surface. (**c**) The fabricated PLLA-GO nanocomposite film (East China University of Science and Technology).

The GO solution prepared in our laboratory was observed and photographed when mixed and after 48 h (Figure 1a). It could be assumed that the GO solution has excellent water dispersibility. This can be explained by the presence of oxygen-containing functional groups on the GO’s surface, including hydroxyl and carboxyl groups as well as other hydrophilic groups [31]. Additionally, it is obvious that AOT/GO exhibits a remarkable dispersibility (Figure 1a). This can be attributed to the capability of AOT to interact with the GO’s surface, leading to hydrophobic properties on the surface of the graphene material [32]. Based on the proposed model of different surfactant/GO interactions from the literature [33,34,35], in Figure 1b, a schematic diagram presents the interaction mode of AOT on the GO’s surface. After the incorporation of GO into the matrix, the film color changed from colorless to black (Figure 1c). The obtained GO filler loading was ~2.4 wt%, which is recognized as an average percentage compared to other works (generally, the amount of GO within the polymers varied from 0.5 to 4 wt%) [15]. 

The composite film’s thickness is ~0.37 mm, which is thicker by about 70 μm compared to neat polymers (PLLA and PLA). The observed increase in the thickness of the composite film compared to neat polymers serves as unambiguous evidence of the GO incorporation into the polymer matrix, as previously reported for other nanoparticle types [36]. The dispersion of GO sheets within the polymer may lead to a certain degree of overlapping or polymer swelling, resulting in a slightly thicker film. GO exerts no substantial influence on the density of PLA, where all samples have an average density of 1.5 g/cm^3^; such values are roughly equivalent to those reported in the open literature [37]. However, it should be noted that the densities of the used PLA and PLLA are slightly higher than most reported values (1.2–1.3 g/cm^3^) [38,39]; this is probably due to the water that was adsorbed on the sample’s surface while the material was handled in air, which is in agreement with the TG analysis (see TG curve below). The photograph of the obtained composite suggests that the GO-based composite displays satisfactory processability as no apparent defects are observed (Figure 1) and it was possible to elaborate square films (80 × 80 cm^2^). 

### 2.1. Morphologic Characterization 

**Figure 2:**
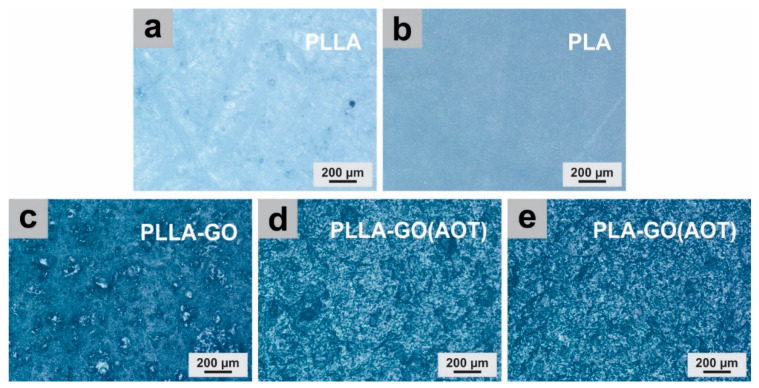
Optical micrographs of neat PLLA and PLA and their obtained nanocomposites, PLLA (**a**), PLA (**b**), PLLA-GO (**c**), PLLA-GO(AOT) (**d**), and PLA-GO(AOT) (**e**). The SEM micrographs of neat polymers and nanocomposite films (PLLA-GO, PLA-GO(AOT), and PLLA-GO(AOT)) are presented in Figure 3 in order to highlight the effect of the GO filler loading and the AOT treatment. No aggregation of GO was observed on the SEM micrographs, indicating good interfacial compatibility. Figure 3b shows that the untreated composite has a smoother and more homogeneous surface compared to the treated ones, indicating that the GO filler does not significantly influence the surface morphology of neat films (Figure 3a). In fact, most studies revealed that PLA has a globally smooth surface [32,39]. When GO was treated with AOT before being used as filler, the nanocomposites exhibited a rough surface (as seen in Figure 3c,d). Moreover, PLLA-GO(AOT) presents a development of many pores compared to the PLA-based composite. The formation of these pores occurred as a result of the entrapment of air within the films during the synthesis process. This morphological structure of PLLA-GO(AOT) could be attributed to the large surface of the GO sheet, which helped air entrapment in the film, and hence, the formation of holes; similar observations were already reported in earlier works [36].

Optical microscopy was used to examine the surface micrographs of both PLA and PLLA and their nanocomposites, aiming to see the GO dispersion in the matrix. The optical micrographs illustrated in Figure 2 show that the GO-based nanocomposites exhibit a dark, textured morphology, while the neat polymer is uniform and transparent. There is not a clear distinction between the matrix and filler, indicating a uniform distribution of GO within the matrix. However, it was noticed that some spots are present on the PLLA-GO surface, which could be linked to the formation of sheet clusters at the microscopic level. On the other hand, as the AOT treatment was used, the fillers appeared to be uniformly dispersed in both the PLA and PLLA matrixes (with no spots observed). This finding is consistent with those of previous reports in the literature [32,40].

**Figure 4:**
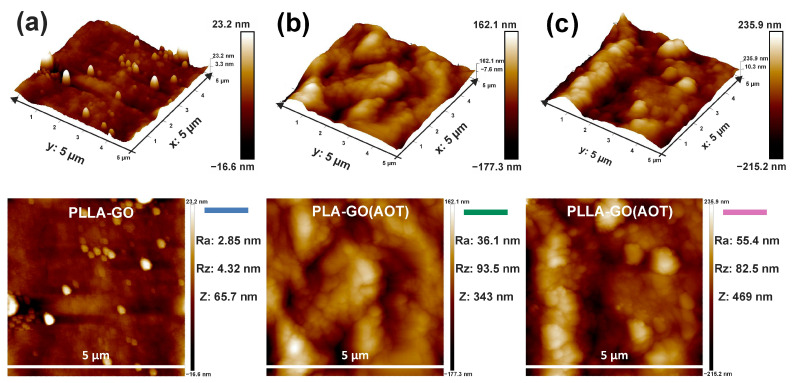
Two-dimensional and three-dimensional AFM images of the obtained nanocomposites: (**a**) PLLA-GO, (**b**) PLA-GO(AOT), and PLLA-GO(AOT) (**c**).

Figure 4 shows the surface morphology and relief of composite films (PLLA-GO, PLLA-GO(AOT), and PLA-GO(AOT)) analyzed via AFM. The treated GO-based nanocomposites (PLLA-GO(AOT) and PLA-GO(AOT)) exhibit a higher topography surface roughness compared to the untreated one (PLLA-GO). The 3D view and 2D topography of the treated GO-based nanocomposites show a surface characterized by hills and valleys, while the untreated film manifests a rather smooth surface. The surface roughness of a sample was determined by analyzing the topography scans of the AFM analysis. The results align closely with those observed in the 2D and 3D studies. In fact, PLLA-GO(AOT) possesses the highest Ra and Rz values of 55.4 and 82.5 nm, respectively, while the PLLA-GO film possesses the lowest values (2.25 and 4.32 nm, resp.). This increased roughness indicates that the treated GO was trapped in the polymer’s matrix, thus increasing the specific surface area of the composite. Also, it could suggest that GO sheets are well dispersed within the matrix [41]. This provides evidence of enhanced dispersion due to the treatment. In contrast, the PLLA-GO surface seems to be smoother, and its morphology is close to the neat PLA. As reported in many papers, it was shown that neat PLA presents a smooth surface compared to filled ones [42,43]. From this result, it is clear that treated GO provides a rough surface to the nanocomposite, which is a characteristic that holds promise for favorable biomedical applications [44]. 

### 2.2. Structural Characterization

#### 2.2.1. FT-IR Analysis

**Figure 5:**
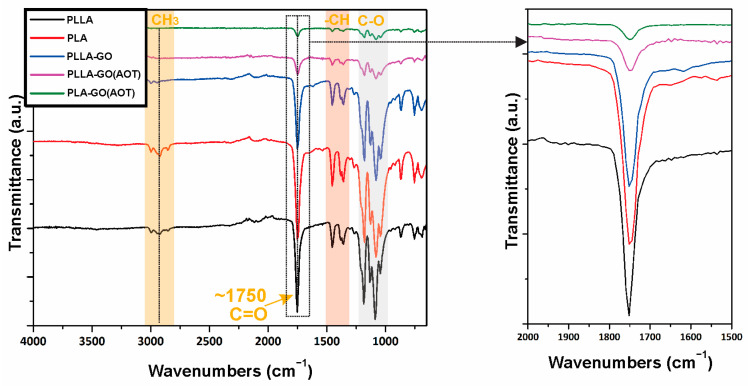
Fourier transform infrared spectra (FT-IR) of neat polymers and their nanocomposites: PLLA, PLA, PLLA-GO, PLLA(AOT)-GO, and PLA(AOT)-GO, respectively.

In order to investigate the interaction between PLA and GO, an FT-IR analysis was undertaken. Figure 5 shows the spectra of the neat polymers (PLLA, PLA) and composite films. The PLLA and PLA samples exhibit similar characteristic peaks at 2996, 2922, 2851, 1750, 1450, 1361, 1182, 1079, 866, and 754 cm^−1^ (Figure 5) because they have the same chemical composition. 

The bands centered at 2996 and 2922 cm^−1^ correspond to the C–H stretching vibration of the CH_3_ groups, while the stretching vibration of the carbonyl group (C=O) is observed at 1748 cm^−1^. The transmittance bands at 1450 and 1361 cm^−1^ are assigned to the symmetric and asymmetric C–H deformations, respectively [45]. The band centered at 1182 cm^−1^ corresponds to the C–O stretching bond in the CH-O groups of PLA and PLLA. These findings are consistent with those of several studies [42,45,46]. 

On the other hand, the FT-IR spectra do not show characteristic peaks of the GO, indicating that PLA is sensitive to infrared rays, which cover the GO peak [27]. A slight shift is observed in the peaks at 1753 cm^−1^ and 1750 cm^−1^ for PLLA and PLA, respectively, by the addition of GO fillers (~2 cm^−1^). This could be attributed to intra-molecular and inter-molecular hydrogen bonding between the filler (GO) and the matrix (PLLA or PLA). Shifting is generally attributed to a strong hydrogen interaction between PLLA or PLA and GO, as reported elsewhere [29,47,48]. Nevertheless, since the resolution was 4 cm^−1^, it was hard to identify such small shifts. Also, the peak localized at 1750 cm^−1^ of composite samples is weak and wide compared with the neat polymers (PLA and PLLA). Moreover, the difference in the peak intensity is more substantial when using treated GO filler (PLA-GO(AOT), and PLLA-GO(AOT)) compared to PLLA-GO, which can be attributed to a positive effect of the AOT treatment by the formation of hydrogen bonds between the GO filler and the matrix [36,49]. This is due to the GO oxygen functional group, which can be linked to functional groups containing oxygen in aliphatic polymers such as PLA, where GO leads to an effective interaction. As a consequence, it can be inferred that GO exhibits excellent interfacial adhesion with polymers [15]. 

#### 2.2.2. XRD Analysis 

**Figure 6:**
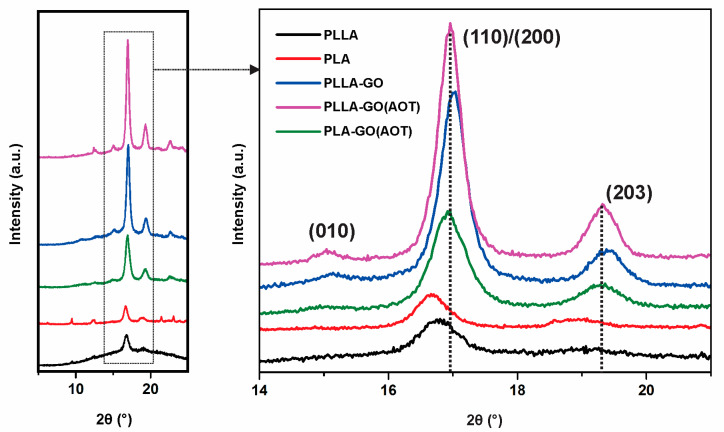
X-ray diffraction patterns of neat PLA and PLLA and their nanocomposites (a colored version of this figure can be viewed online).

XRD analyses were conducted at room temperature to investigate the crystallization of the neat polymers and the nanocomposites, shedding light on the intercalation and exfoliation of GO in the PLA matrix. The XRD patterns of PLLA, PLA, PLLA-GO, PLLA-GO(AOT), and PLA-GO(AOT) films are compared in Figure 6. The characteristic peak of GO (2 θ = 11.6°) observed in several studies is not present in any of the prepared nanocomposites. This suggests that the layered GO was efficiently exfoliated into single or few layers of stacked platelets and was fully dispersed throughout the PLA matrix [50]. The neat PLLA and PLA have their strongest peaks at 16.7° and less intense peaks at about 18.9 and 22.1°, which were attributed to reflections of the (200)/(110), (203), and (015) reticular planes, respectively. The XRD analysis of PLA and PLLA corroborates the information reported in the previous literature, indicating that the peaks at 16.7°, 18.9°, and 22.1° correspond to the α-crystalline phase of PLA crystallizing in an orthorhombic symmetry [28,32,51]. Also, the shapes of the PLA and PLLA XRD patterns indicate that the matrix morphology presents some degree of an amorphous phase [42,46,50]. The nanocomposites have similar XRD patterns to those of the neat PLLA and PLA (all PLLA and PLA with characteristic peaks are observable in the composites, indicating that the addition of GO did not significantly modify the crystal structure of poly(lactic acid) in the nanocomposites). 

The findings indicate that the crystallization rate of composite films is higher compared to neat PLA and PLLA. Moreover, the peak intensity increases significantly after AOT treatment, indicating that GO improves the crystallinity of poly(lactic acid), and the treatment enhances the GO/matrix interaction, resulting in further improved crystallization [14,28]. Similarly, Sun et al. indicated that well-dispersed covalently bonded GO sheets induce a heterogeneous nucleating effect. As a result, there is a decrease in the activation energy for crystallization, leading to an increase in the crystallinity of GO-based nanocomposites compared to pure PLA [25]. Remarkably, the XRD data reveal that the 2θ values for the (110/200) reflection are slightly shifted from 16.74 for PLLA to 17.03° for PLLA-GO, and 16.95° for PLLA-GO(AOT). Likewise, the 2θ° values are shifted from 16.66° for PLA to 16.92° for PLA-GO(AOT) (Figure 6). This shift suggests a slight decrease in the *d*-spacing between the reticular planes within the crystal structures of the composite with the introduction of GO fillers. Also, the slight difference between the treated and untreated GO composite could be attributed to the variable hydrophilicity of the fillers (treated and untreated GO) [32]. For instance, Li et al. found that a GO with a silane coupling agent can act as a heterogeneous nucleating site for a PLA/GO nanocomposite [14]. The capacity of fillers to act as nucleation centers for the crystallization of polymers depends on their dispersion and their interfacial adhesion with the matrix [50]. The results cited above suggest that the AOT treatment led to well-dispersed (non-aggregated) GO fillers with suitable filler–matrix interfacial adhesion.

### 2.3. Thermal Properties

#### 2.3.1. DSC Analysis 

**Figure 7:**
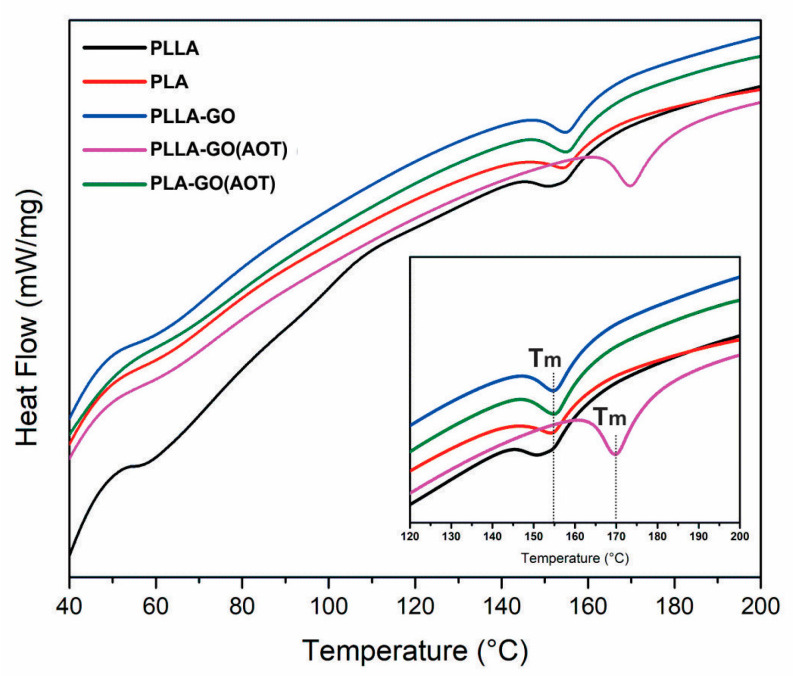
DSC thermograms of neat PLA and PLLA and their nanocomposites.

The thermal behavior of the samples was investigated using DSC analysis (Figure 7). The unimodal endotherm peak suggests a homogeneous distribution of crystals with a uniform thickness, which is attributed to the melting of stable crystals of PLLA and PLA. In addition, the GO filler does not significantly affect the temperature T_g_ of neat polymers (Figure 7). This corroborates previous works, thus indicating that GO has no influence on the formation of PLA chain molecules [28,50]. On the other hand, introducing GO to the PLLA matrix results in a shift of T_m_ toward a higher temperature. Additionally, the AOT-treated composite (PLLA-GO(AOT)) exhibits a further displacement. In fact, the DSC profiles clearly show that adding GO fillers treated with AOT increases the temperature T_m_ of PLLA by 17 °C and gives an enhancement compared to the untreated composite of 15 °C. Moreover, increases in the melting peak intensity are observed upon the incorporation of the GO filler into the PLLA matrix. Furthermore, more enhancement occurred when using AOT treatment, which is attributed to the increased crystallinity of the nanocomposites [52]. However, the GO filler did not significantly increase the melting peak intensity of PLA. This could be expanded by the low increase in crystallinity due to the addition of GO to PLA (see Figure 6 DRX). These results match well with the XRD analysis. 

It was shown that GO treatments enhance their compatibility with the PLA matrix, thereby facilitating the nucleation of PLA crystallization [32,50]. This suggests that GO could act as a nucleating agent when it is well adhered to the polymer, which lowers the critical nucleus size that is necessary for the development of a thick and stable nucleus in PLA [28]. Finally, it can be deduced that there is a positive correlation between the increase in crystallinity and the corresponding elevation in the melting temperature (T_m_).

#### 2.3.2. TGA and DTG Analysis 

**Figure 8:**
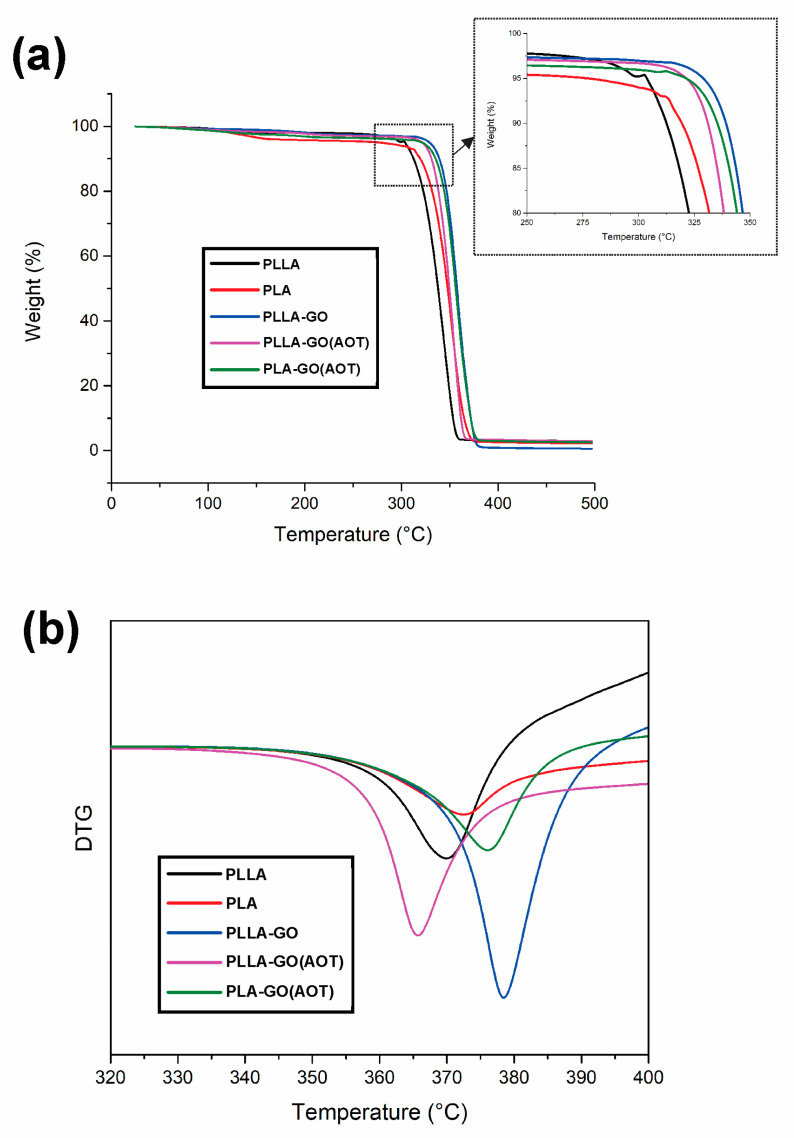
Thermal characterization of neat PLA and PLLA and their nanocomposites. (**a**) TGA and (**b**) DTG curves.

Thermal stability is a critical property in the development of PLA-based materials. Hence, the developed materials were evaluated using thermogravimetric analysis (TGA), and the main results are presented in Table 1. Figure 8a illustrates the results of the TGA analysis, which displays the weight loss versus temperature curves for both the neat polymers and nanocomposites. The initial stage of the analysis indicates that all films underwent a degree of weight loss, which is potentially attributed to the evaporation of water that is present within the sample. This water’s presence may contribute to an increased density of the samples compared to the values reported in the literature (please refer to the above paragraph). 

With the exception of the PLA, all other samples remain stable without significant weight loss up to ~280 °C. The weight loss stage occurs between 280 and 400 °C, while in the temperature range of 100–200 °C, the PLA displays a weight loss of 4%, which is significantly higher than the other samples; the homemade PLLA presents better stability in this temperature range. As mentioned in the literature, this weight loss is mainly attributed to the evaporation of physisorbed water and other impurities [29,53]. When temperatures exceed 300 °C, the main thermal degradation process starts with the acceleration of weight loss. The process is mainly attributed to intra-molecular transesterification (backbiting reaction) [28,54].

The onset temperature (T_onset_) corresponds to a weight loss of 10%, which is equal to 312.5 and 319.9 °C for PLLA and PLA, respectively. On the other hand, the T_onset_ is found to be 338.9, 331.1, and 335.5 for the PLLA-GO, PLLA-GO(AOT), and PLA-GO(AOT) nanocomposites, respectively. The decomposition temperatures at a 50% weight loss (T_D1/2_) for the neat polymers are 337.9 °C for PLLA and 347.6 °C for PLA. For the nanocomposites PLLA-GO, PLLA-GO(AOT), and PLA-GO(AOT), the decomposition temperatures are 357.3, 349.5, and 356.1 °C. In fact, the temperatures (50% weight loss) for the PLLA-GO and PLA-GO(AOT) nanocomposites increased by 19.4 and 8.5 °C compared to neat PLLA and PLA, respectively. The following maximum decomposition temperatures (T_max_) are determined from the curves: PLLA: 359.3 °C; PLA: 373.3 °C; PLLA-GO: 380.2 °C; PLLA-GO(AOT) 367.4 °C; and PLA-GO(AOT): 378.7 °C. 

The results show that the decomposition temperatures increase by about 21 and 5.4 °C for the PLLA and PLA, respectively, when using GO. It is clear that the GO fillers enhance the thermal stability of both PLLA and PLA. The obtained results are well corroborated by other works [27,53]. This enhancement is attributed to interfacial interactions between polymers and GO by hydrogen bonds and Van der Waals forces [43,51]. However, when using an AOT treatment, the thermal stability of the PLLA-based composite decreases only by 12 °C, indicating a negative effect of the chemical crosslinking treatment compared to an untreated specimen. But this treatment did not deteriorate the stability of the neat polymer (an improvement of 8 °C). 

The temperatures at the maximum degradation rate (T_D_) are determined from the derivative thermogravimetric (DTG) curves (Figure 8b) as follows: PLLA: 369.8 °C; PLA: 372.4 °C; PLLA-GO: 378.4 °C; PLLA-GO(AOT): 365.7 °C; and PLA-GO(AOT): 376.3 °C. All samples exhibit a single peak, demonstrating that the degradation occurs in only one step (Figure 8b). The results show that the decomposition temperature increases by 8.6 °C and 4 °C for the PLLA and PLA, respectively, when using GO. These results are consistent with the previous findings reported by others [28,49]. According to Valapa et al. [28], the observed enhancement can be ascribed to the barrier effect exerted by graphene, which limits heat transmission. Globally, the TGA experiment’s positive outcome corroborates the DSC analysis results. Hence, it can be concluded that GO improves the thermal stability of PLLA. Moreover, the surface treatment of GO gives the event more improvement. 

### 2.4. Water Contact Angle 

**Figure 9:**
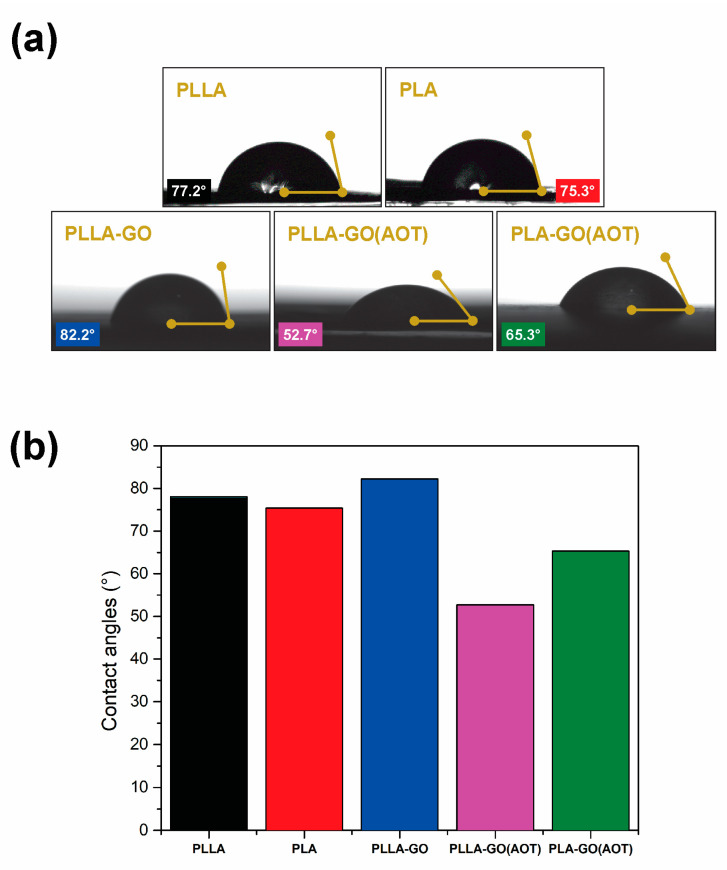
Water contact angle characterization. (**a**) Water droplets on neat polymers and their nanocomposites, and (**b**) histogram of water contact angle values.

The wettability of PLLA and PLA and their nanocomposites was studied through a water contact angle test. Figure 9a shows images of water droplets on the sample surfaces, and Figure 9b presents a histogram of the measured contact angles. The results reveal that PLLA exhibits a slightly higher WCA (77.2°) compared to PLA (75.3°). When adding untreated GO, the WCA of the PLLA sample increases slightly, which means that the wettability of the resulting composite remains almost unchanged. On the other hand, when using the treated GO, the results reveal a decrease in the contact angle (52.7 and 65.3 for PLLA-GO(AOT) and PLA-GO(AOT), respectively). It can be noted that treated GO improves the hydrophilicity of PLLA and PLA, which provides more wettability to the composite. Indeed, the WCA of PLLA-GO(AOT) decreases drastically by 28° compared with neat PLLA. These results are consistent with those of previous studies [24,55] and could be attributed to the high polarity of GO associated with its hydrophilic functional groups [44,56,57]. Additionally, it was demonstrated that the surface wettability can be influenced by varying crystallinity [58]. The decrease in the contact angle of PLLA-GO(AOT) compared to PLLA and PLLA-GO could be attributed to differences in crystallinity.

On the other hand, the contact angle can be influenced by various factors, including surface energy, which is determined by the chemical composition and structure of the surface. Consequently, the chemical modification employing AOT leads to surface functionalization through the introduction of anionic groups. These groups directly alter the contact angle and wettability, resulting in increased hydrophilicity. The contact angle can also be influenced by the surface roughness and pores, which can create additional contact sites with the surface. This observation is supported by the analysis of the SEM and AFM micrographs, which demonstrated the notable impact of roughness and pores on the alteration of contact angles. Hydrophilicity, as a surface property, has a significant impact on the biological characteristics of biomaterials, which affecst protein adsorption and cell adhesion [44]. In this context, the hydrophilicity of PLLA-GO(AOT) is desirable for promoting cell adhesion and proliferation, which can be applied in tissue engineering as a biomaterial scaffold [24]. 

## 3. Materials and Methods

### 3.1. Material: Component Overview 

The selected matrix was a poly(lactic acid), recognized as a biocompatible and biodegradable polymer that is widely used in medical devices. Two variants were used: The first one was a commercial poly(lactic acid) (PLA), purchased from Shanghai Chemical Reagent Co., Ltd. (Shanghai, China). The second one was a homemade polymer (PLLA) synthesized in the Key Laboratory for Ultrafine Materials of the Ministry of Education (East China University of Science and Technology, Shanghai, China). The used filler consisted of graphene oxide (GO), synthesized by the oxidation of graphite flakes (325 mesh) supplied by Anhui Zesheng Technology Limited Company (Shanghai, China). The oxidation process involved the use of potassium permanganate from Sinopharm Chemical Reagent Co., Ltd., Shanghai, China and aqueous HCl from Titan Hydrochloric Acid Technology Co., Ltd., Shanghai, China. The surface treatment of graphene involved the utilization of an anionic surfactant known as dioctyl sulfosuccinate sodium salt (AOT), which was procured from Shanghai Macklin Biochemical Co., Ltd., Shanghai, China. The biocomposite was prepared using two solvents: DCM purchased from Jiangsu Anway Chemical Technology Co., Ltd., Changzhou, China and DMF from Shanghai Macklin Biochemicals Co., Ltd., Shanghai, China. 

### 3.2. Synthesis of GO

Due to its simplicity and scalability, the modified Hummers method is widely used to synthesize graphene oxide [16]. In this method, graphite flakes are oxidized with a mixture of potassium permanganate (KMnO_4_) and sulfuric acid (H_2_SO_4_). The reaction was carried out under controlled conditions, including temperature and time. The oxidation introduces oxygen-containing functional groups onto the graphene surface, resulting in the formation of GO. These functional groups, such as hydroxyl and carboxyl groups, enable the dispersibility of GO in various solvents, thus facilitating chemical modifications. Hence, in this work, GO was prepared from graphite flakes following a modified Hummer’s method as follows: 3 g of KMnO_4_ was poured in 23 mL of H_2_SO_4_ in a Pyrex beaker in an ice bath. As a result, the solution’s color turned green. To ensure the dispersion, 1 g of graphite was gradually added to the solution at 35 °C; the resulting mixture was stirred for 1 h. Subsequently, 50 mL of DI water was added, and the solution’s color turned brown, followed by heating at 85 °C in an oil bath for 15 min. Under an ice bath, 10 mL of hydrogen peroxide (H_2_O_2_) was added. The color of the solution turned earthy yellow, indicating a successful interaction. Using HCl solution (1.2 M), GO was washed via suction filtration in acetone and then underwent suction filtration again. After the synthesis process, the GO suspension was allowed to dry, resulting in the formation of powdered GO.

### 3.3. Specimen Preparation

A homogeneous dispersion of GO (50 mg) with AOT (2 mg) was prepared via sonication in dimethylformamide (DMF) with the formula (CH_3_)_2_NC(O)H (10 mL) for 30 min at room temperature. AOT was added to achieve a uniform dispersion of GO sheets in the matrix material [34]. A total of 2 g of PLA or PLLA was dispersed into 50 mL of Dichloromethane (DCM, CH_2_Cl_2_). The mixture of the modified GO was added to dissolved PLA or PLLA under vigorous magnetic stirring for 1 h. AOT-treated GO further reacted with PLA to form LA-linked GO dispersed within a PLA matrix. The obtained solution was left to dry in a mold at room temperature for 24 h, inside a fume hood. The obtained composite was hot-pressed using a flat vulcanizing machine (DONGGUAN BOLON PRECISION TESTING MACHINES, Dongguan, China). The used flat vulcanizing machine provides uniform pressure distribution, heat transfer, control, precision, and easy loading and unloading, resulting in high-quality finished products and reliable manufacturing processes. Three nanocomposites were prepared using treated and untreated GO as fillers and PLA and PLLA as matrixes. PLLA filled with untreated GO, PLLA filled with treated GO, and PLA filled with treated GO samples were labeled PLLA-GO, PLLA-GO(AOT), and PLA-GO(AOT), respectively. They were compared to both neat PLLA and PLA polymers.

### 3.4. Characterizations

The surface morphology of the prepared materials was examined via scanning electron microscopy (SEM; ZEISS Sigma 300 VP, Carl Zeiss, Oberkochen, Germany). A carbon spray (Aerodag^®^ G, Acheson Industries Inc., Port Huron, MI, USA) was used to treat the samples in order to make them suitable for observation. Using an atomic force microscope (AFM) (Dimension Icon AFM, Bruker, Billerica, MA, USA), high-resolution 3D pictures of the surface morphology were obtained; it operated in tapping mode with a scan range of 50 × 50 μm^2^, and the images were analyzed using AFM-nanoscope software. The FT-IR spectra were plotted with a Cary 630 FTIR Spectrometer (Agilent Technologies, Inc., Santa Clara, CA, USA) with a resolution of 4 cm^−1^ in the range 650–4000 cm^−1^; this was performed to track changes in the chemical structure of the samples. A Bruker diffractometer was employed to conduct X-ray diffraction (XRD) analysis (Bruker D2 PHASER, Bruker, Billerica, MA, USA). The analyses were carried out at a voltage of 40 kV and a current of 30 mA using monochromatic Cu-Kα radiation (λ = 0.154 nm); the measurements were taken in the 2θ range (5–60°) with a scanning rate of 1°/min. 

The glass transition temperature (T_g_) and melting temperature (T_m_) were determined using differential scanning calorimetry (DSC; TA Instruments, Inc., New Castle, DE, USA). About 8 mg of each sample was subjected to a heating rate of 10 °C/min from room temperature up to 200 °C. High-purity aluminum was used as a reference sample. The thermal stability was determined using thermal gravimetric analysis (TGA). Hence, the temperature-dependent weight loss measurements were carried out using a thermo-gravimetric analyzer (DSC-ATG SDT 600, supplied by TA Instruments, New Castle, DE, USA) under N_2_ atmosphere from 25 to 600 °C at a heating rate of 10 °C/min. The samples were held at 600 °C for 5 min and subsequently cooled to room temperature. 

To assess the surface’s wettability, water contact angle (WCA) measurements were carried out by depositing 3 μL drops of EDI-deionized water on the films; a contact angle meter instrument was used for this purpose (Digidrop, GBX, Romans-sur-Isere, France). The droplet photos were captured using a built-in digital camera equipped with Visiodrop software. The results represent the average of three or more measurements. 

## 4. Conclusions

In this study, nanocomposite films were successfully prepared based on PLLA and PLA matrixes filled with treated and untreated graphene oxide (GO). An easy and fast method was issued to produce homogenous materials for potential use as biomedical devices. Several properties of PLLA and PLA were enhanced when filled with GO. Interestingly, the AOT surface treatment of GO gives a remarkable improvement. The results show that the prepared composite films exhibited uniform GO sheet dispersion and had good interfacial compatibility with the matrix. Indeed, the effective surface treatment of GO fillers leads to enhanced crystallinity, hydrophilicity, and thermal properties of the resulting nanocomposites. 

The FT-IR results indicate the formation of bonding between the matrix and the fillers. Additionally, the crystallinity of the polymers was enhanced by the addition of GO with AOT treatments, resulting in a significant increase in the peak intensity. The thermal properties were also improved; according to DSC thermograms, incorporating AOT-treated GO fillers into the PLLA composite increased the melting temperature (T_m_) by 17 °C. Similarly, the TGA results confirmed a significant increase in the decomposition temperatures of PLLA by 21 and 8 °C upon using GO and AOT-treated GO, respectively. Furthermore, the contact angle tests indicated a positive wettability of the PLLA-GO(AOT), which can potentially improve cell adhesion in tissue engineering. 

The findings provided in this study have the potential to significantly improve the performance of nanocomposites, which have emerged as a promising choice for applications in the field of biomedical engineering. Furthermore, this treatment for GO can even be applied to improve other biopolymers. In perspective, to further assess the effect of such treatment, mechanical and biological testing should be conducted to confirm the performance of this nanocomposite as a biomaterial. Furthermore, varying the percentage of GO and the treatment could help to optimize these nanocomposites.

## Figures and Tables

**Figure 3:**
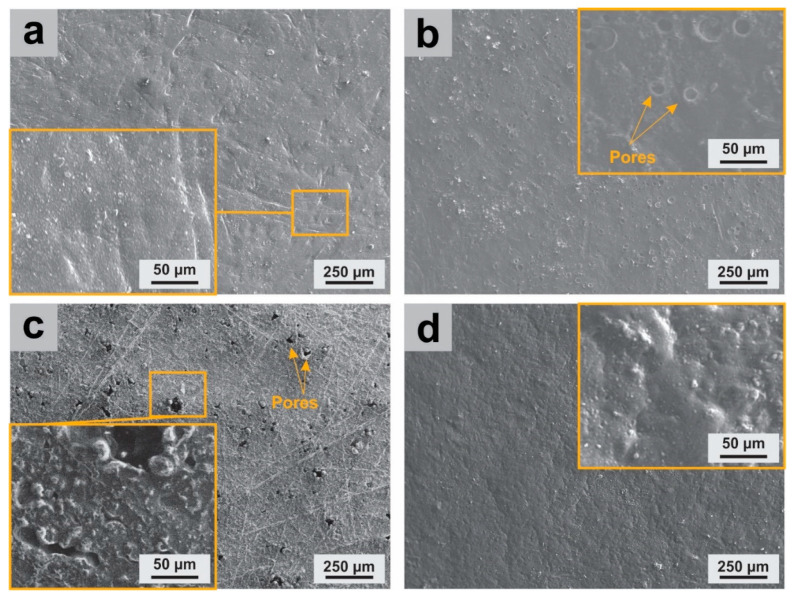
SEM micrographs of neat PLLA and the obtained nanocomposites, PLLA (**a**), PLLA-GO (**b**), PLLA-GO(AOT) (**c**), and PLA-GO(AOT) (**d**).

**Table 1:** Summary of TGA results.

Sample	Thermal Decomposition Temperatures		
	Starting (10% Loss)	(50% Loss)	Maximum
PLLA	312.5	337.9	359.3
PLA	319.9	347.6	373.3
PLLA-GO	338.9	357.3	380.2
PLLA-GO(AOT)	331.1	349.5	367.4
PLA-GO(AOT)	335.5	356.1	378.7

## Data Availability

The data that support the findings of this study are available from the corresponding authors upon reasonable request.
